# Association of multiple ischemic strokes with mortality in incident hemodialysis patients: an application of multistate model to determine transition probabilities in a retrospective observational cohort

**DOI:** 10.1186/s12882-016-0350-3

**Published:** 2016-09-21

**Authors:** James B. Wetmore, Jonathan D. Mahnken, Milind A. Phadnis

**Affiliations:** 1 Division of Nephrology, Hennepin County Medical Center, 701 Park Avenue, Minneapolis, MN 55415 USA; 2 Department of Biostatistics, University of Kansas School of Medicine, Kansas City, KS USA

**Keywords:** Stroke, Mortality, Dialysis, ESRD, Medicare, USRDS

## Abstract

**Background:**

Little is known about the effect of multiple, or subsequent, ischemic strokes in patients receiving hemodialysis.

**Methods:**

We undertook a retrospective cohort study of incident hemodialysis patients with Medicare coverage who had experienced a first ischemic stroke. Factors associated with either a subsequent ischemic stroke or death following a first new stroke were modeled. A multistate model with Cox proportional hazards was used to predict transition probabilities from first ischemic stroke to either subsequent stroke or to death, and the demographic and clinical factors associated with the respective transition probabilities were determined. Effect of a subsequent ischemic stroke on survival was quantified.

**Results:**

Overall, 12,054 individuals (mean age 69.7 years, 41.3 % male, 53.0 % Caucasian and 34.0 % African-American) experienced a first new ischemic stroke. Female sex was associated with an increased risk of having a subsequent ischemic stroke (adjusted hazard ratio 1.37, 95 % confidence intervals 1.20 – 1.56, *P* < 0.0001); African-Americans, as compared to Caucasians, had lower likelihood of dying after a first new ischemic stroke (0.81, 0.77 – 0.85, *P* < 0.0001). A subsequent stroke trended towards having a higher likelihood of transitioning to death compared to a first new ischemic stroke on dialysis (1.72, 0.96 – 3.09, *P* = 0.071). When a subsequent ischemic stroke occurs at 24 months, probability of survival dropped >15 %, in absolute terms, from 0.254 to 0.096, with substantial drops observed at subsequent time points such that the probability of survival was more than halved.

**Conclusions:**

Likelihood of subsequent ischemic stroke and of survival in hemodialysis patients appears to vary by sex and race: females are more likely than males to experience a subsequent ischemic stroke, and Caucasians are more likely than African-Americans to die after a first new ischemic stroke. The risk of a transitioning to a subsequent stroke (after having had a first) increases until about 1 year, then decreases. Subsequent strokes are associated with decreased probability of survival, an effect which increases as time since first stroke elapses. This information may be of assistance to clinicians when counseling hemodialysis patients about the implications of recurrent ischemic stroke.

## Background

Stroke, a catastrophic health event with profound implications for morbidity and mortality, impacts both the individual and society as a whole [1]. Like virtually all cardiovascular events, stroke is especially common in patients receiving chronic dialysis, with many reports having helped to quantify the incidence and prevalence of stroke [2–8], its geographic variation in the U.S. [9], and its association with mortality and years of life lost [10].

However, there is little information about multiple, or repeat, ischemic strokes in hemodialysis (HD) patients. Individuals who experience multiple strokes might differ in substantial ways from those who only ever have a single stroke, and the clinical implications of a repeat, or subsequent, stroke on mortality is unknown. To ascertain how often subsequent ischemic strokes occur in HD patients, which factors might be associated with subsequent strokes, and how such strokes are associated with mortality, we constructed a large cohort of incident HD patients using data from the United States Renal Data System (USRDS) and Medicare. We employed a novel multi-state modeling approach to investigate how demographic characteristics might be associated with subsequent strokes and post-stroke survival, and how the risk of death after a first ischemic stroke compared to risk of death after a subsequent one. We hypothesized that the hazard ratio for mortality would increase for a subsequent, as compared to an initial, ischemic stroke. Findings in this area could ultimately improve the understanding of stroke-related mortality in patient receiving HD, provide data with which to counsel patients, and better guide future stroke-awareness and -prevention efforts among patients, healthcare providers, and the dialysis community.

## Methods

### Study design, cohort, and data sources for analysis

We performed a retrospective cohort analysis of incident, chronic HD patients who were >18 years of age, initiated dialysis on or after January 1, 2000 through October 2, 2005, were continuously enrolled in Medicare, had a minimum of 90 days of follow-up after cohort inclusion, and who had had a stroke on or after day 91 of HD. Medicare, a federally-funded program for which nearly all adults with end stage renal disease are entitled, insures the vast majority of chronic dialysis patients [2, 11]. Patients enrolled in managed care plans or in the Department of Veterans Affairs health system were excluded.

Data for these analyses were from the USRDS, a national system that collects data on virtually all patients undergoing chronic dialysis in the U.S. From the USRDS, we received standard patient records that included demographics and comorbidities at the time of dialysis commencement. The USRDS also incorporates data on inpatient and outpatient medical claims paid by Medicare, which contain International Classification of Diseases – 9th Revision (ICD-9) codes for each date of service.

To assure that we were studying chronic HD patients and to help establish the presence of comorbidities, patients were required to initially survive 90 days; first new strokes could therefore occur, at the earliest, on day 91. All patients who had a first new stroke then constituted the analytic cohort, or “risk pool”, for subsequent stroke and death events. Individuals were then censored upon loss of Medicare coverage, or receipt of a kidney transplant on or before December 31, 2005.

### Covariates and descriptive variables

Demographic and clinical variables were drawn from the CMS 2728 Medical Evidence Form and supplemented by Medicare claims. These included age, sex, race by ethnicity, body mass index, employment status, smoking, substance abuse (alcohol or illicit drugs), inability to ambulate and to transfer, cause of ESRD, and comorbidities. Age was treated as a time-dependent covariate (i.e., it was assessed at first stroke and again later at second stroke, if it occurred). Race/ethnicity was categorized into one of four mutually exclusive groups: non-Hispanic Caucasians, non-Hispanic African-Americans, Hispanics, and Others. Body mass index (BMI) was classified into 4 categories: < 20 kg/ m^2^, 20–24.99 kg/m^2^, 25–29.99 kg/m^2^, ≥ 30 kg/m^2^. Cause of ESRD was categorized as diabetes, hypertension, glomerulonephritis, or other. Because the CMS 2728 form is structured such that diabetes and hypertension may be considered as both a cause of ESRD and/or a “freestanding” comorbidity, for the purposes of the present analysis, these two covariates were considered a comorbidity if they were listed as either the cause of ESRD or as a freestanding comorbidity on the CMS 2728 form [12]. Initial comorbidities, ascertained at dialysis initiation from the CMS 2728 form, consisted of diabetes, congestive heart failure, coronary artery disease, cerebrovascular disease, and peripheral vascular disease. We did not censor patients if they changed to peritoneal dialysis.

### Stroke identification

We utilized recent information on the sensitivity and specificity of stroke-related ICD-9 claims [13] to identify ischemic strokes from Medicare data, as has been done previously by Go et al. [9, 14] Briefly, a patient was considered to have an ischemic stroke if the principal diagnosis ICD-9 code at the time of hospital discharge was 434 or 436 and one of the following occurred: (a) the patient expired during the hospitalization; (b) the hospitalization lasted ≥ 48 h; or (c) the hospitalization lasted < 48 h and the patient did not have a carotid endarterectomy (ICD-9 code 381.2). In the absence of 434 or 436, ICD-9 code 362.3 was sufficient to diagnose an ischemic stroke. To establish the analytic cohort (risk pool), all individuals had to experience a first new stroke on or after day 91 of dialysis (i.e., after having survived the first 90 days of dialysis). Subsequent strokes were not only identified using the same complex algorithm, but also had to be separated by at least 30 days from a previous stroke event, in order to reduce the likelihood of misclassification resulting from a readmission for a previous stroke. No minimum time was established between stroke and death; the latter could occur immediately as a result of the former. Causes of death were not considered; we sought only to determine the relationship between stroke and subsequent all-cause mortality.

### Statistical analyses

We generated descriptive statistics to illustrate how individuals who experienced subsequent ischemic stroke differed from those who experienced a first ischemic stroke. Bivariate analyses comparing each of the explanatory variables were performed using Pearson’s chi-squared test or Student’s *t*-test, as appropriate.

**Fig. 1:**
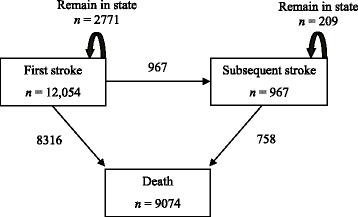
The multistate model and transitions of the participants

We modelled survival following a first or subsequent ischemic stroke using a multistate modeling approach. (Thus, by definition, all studied individuals had to experience a first stroke, which constituted “time zero”.) The multistate modeling approach permits modeling of the hazard of transitioning from one state to the next possible state using the familiar Cox proportional hazards (PH) framework. Specifically, with this approach, an individual is conceptualized as being in one of the three possible states: alive after having had only a first stroke; alive after having had a subsequent stroke; or dead. Accordingly, there are three possible types of accompanying transitions (Fig. 1), with censoring possible in any of these three transitions and modeling in each of these transitions is done using the Cox PH model. The advantage of using this approach is that for states that transition to the terminal outcome (*i.e.*, death), the Cox PH model can be used to estimate the hazard ratio of death for experiencing a subsequent stroke compared to experiencing only the first stroke after adjusting for the effect of clinically significant covariates specific to that transition. In this context, subsequent stroke is treated as a time-dependent covariate, *i.e.*, it may or may not occur at some time after first stroke *en route* to death [15–17]. Equivalence between a multi-state model with a single intermediate state (such as ours) as a Cox model with a single time-dependent covariate has been illustrated by Putter et al. [15] We explicitly tested whether the assumptions of the proportional hazards modeling approach were valid by including an additional term assessing the “effect” of subsequent stroke over time (i.e., testing for a stroke-by-time interaction), but, finding this to interaction to be nonsignificant, we reported the more parsimonious model.

One advantage of this approach, which has been used to investigate clinical issues previously [16], is that it allows the other baseline risk factors to have different (or “freely varying”) effects in each transition, which is theoretically appealing because it is possible that baseline comorbidities may play a different role in likelihood of mortality based on whether a patient has experienced only one stroke or more than one stroke. In the case of a relatively small number of subsequent events (such as subsequent strokes, which were pooled from second and third strokes), this approach also permits the flexibility to fit parsimonious models for the baseline covariates in each transition and defends against model over-fitting. This is done by first fitting a full model with all covariates (in each transition) and then sequentially deleting those covariates that are not found to be statistically significant. This process is continued until a parsimonious model that includes only statistically significant predictors is generated. By appropriately combining the estimated baseline hazards and the regression coefficients it is possible to calculate the (predicted) transition probability of moving from state “A” to state “B” in time interval (s, t] as outlined in Klein et al. [18] This is done by first estimating the baseline cumulative hazard, adjusting it for the effect of covariates, and using it to estimate survival probabilities in each transition. These estimated survival probabilities are then used to estimate the (predicted) transition probabilities by using the appropriate integration formulas. For example, in the case of moving from “first stroke” to “subsequent stroke”, the transition probability is the probability of experiencing a recurrent stroke in interval (0, t] given that the patient starts at time = 0 with one stroke. For the case of moving from “first stroke” to “death”, it is the probability of dying in interval (0, t] given that the patient started at time = 0 with one stroke. (That is, a patient could die directly after only one stroke in this interval or die via experiencing second stroke.) This approach facilitates calculation of predicted probabilities of being in any given state at any time after cohort entry and permits clinically meaningful inferences to be made. As Cox PH models are used in each transition, the proportionality of hazards assumption for all the categorical predictors in the model was assessed by means of a log-log survival plot. In addition to being able to incorporate the time-dependent nature of a events such as a subsequent stroke, this method allows the flexibility in the use of baseline risk factors affecting the likelihood of mortality differently in each transition. Parsimonious models (in terms of covariates) can be fit, while use of the Cox proportional hazards framework facilitates calculates probabilities of transition from the current state to the next state within a given time interval.

For all analyses, *P* < 0.05 was considered statistically significant. The 95 % confidence intervals were obtained using Wald formulas. All statistical analyses utilized R statistical software, with the “mstate package” being used specifically for the main analyses [17].

At all times, we adhered to the STrengthening the Reporting of OBservational studies in Epidemiology (STROBE) guidelines in the design, analysis, and reporting of this study.

### Compliance and protection of human research participants

The research protocol was approved by the institutional review board at the University of Kansas Medical Center (KUMC). Data Use Agreements (DUA) between KUMC and the USRDS and CMS were in place.

## Results

### Cohort characteristics

**Fig. 2:**
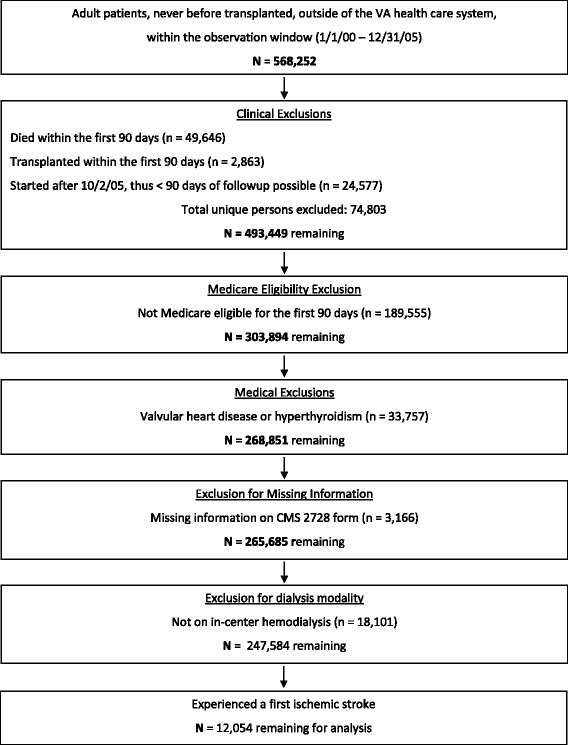
Construction of the analytic cohort

Figure 2 shows the construction of the cohort. There were 12,054 individuals who satisfied all inclusion criteria and who experienced a new stroke on or after day 91 of hemodialysis, thereby forming the analytic cohort. Mean age was 69.7 years, 41.3 % were male, and 53.0 % were Caucasian while 34.0 % were African-American.

Of the 12,054 who experienced a first stroke, 2771 (23.0 %) were alive at time of censoring (“remained in state”) a mean of 12.5 ± 13.8 months after their stroke; 967 (8.0 %) transitioned to a subsequent stroke a mean of 10.3 ± 9.9 months after their first stroke, and 8316 (69.0 %) died a mean of 9.8 ± 12.0 months their stroke (Fig. 1). Of those who experienced a subsequent stroke, 78.4 % subsequently died a mean of 7.8 ± 9.9 months after the subsequent stroke.

Persons were divided into those who experienced one stroke and those who experienced > 1 stroke; their characteristics are shown in Table 1. In general, individuals who experienced > 1 stroke were marginally younger, more likely to be female, more likely to be African-American (compared to Caucasian), more likely to have diabetes, and more likely to have had a previous stroke before entering the cohort and to have permanent atrial fibrillation.

**Table 1:** Descriptive characteristics of the dually-eligible cohort, by stroke status

Characteristic^a^	One Stroke Only	>1 Stroke
No. of persons	11087	967
Age, yr	69.8 (11.8)	68.4 (10.8)
Male	4640 (41.9 %)	342 (35.4 %)
Race/Ethnicity		
African-Amer.	3718 (33.5 %)	376 (38.9 %)
Caucasian	5912 (53.3 %)	471 (48.7 %)
Hispanic	1063 (9.6 %)	92 (9.5 %)
Other	394 (3.6 %)	28 (2.9 %)
BMI category		
< 20 kg/m^2^	1170 (10.6 %)	83 (8.6 %)
20 –24.9 kg/m^2^	3696 (33.3 %)	297 (30.7 %)
25 –29.9 kg/m^2^	3197 (28.8 %)	312 (32.3 %)
≥ 30 kg/m^2^	3024 (27.3 %)	275 (28.4 %)
Smoker	500 (4.5 %)	40 (4.1 %)
Substance abuser	137 (1.2 %)	14 (1.5 %)
Unemployed	10924 (98.5 %)	948 (98.0 %)
Unable to ambulate	659 (5.9 %)	55 (5.7 %)
Unable to transfer	261 (2.4 %)	23 (2.4 %)
Hb < 11.0 g/dL^b^	7540 (68.0 %)	652 (67.4 %)
Comorbidities		
HTN	9668 (87.2 %)	856 (88.5 %)
DM	6794 (61.3 %)	645 (66.7 %)
CHF	4324 (39.0 %)	352 (36.4 %)
CAD	3715 (33.5 %)	303 (31.3 %)
PVD	2066 (18.6 %)	189 (19.5 %)
Prior CVA	4429 (40.4 %)	533(55.1 %)
Permanent AF	2470 (22.3 %)	229 (23.7 %)
Cause of ESRD		
DM	6014 (54.2 %)	584 (60.4 %)
HTN	3019 (27.2 %)	233 (24.1 %)
GN	500 (4.5 %)	35 (3.6 %)
Other	1554 (14.0 %)	115 (11.9 %)

*Abbreviations*: *African-Amer* African-American, *BMI* body mass index, *Hb* hemoglobin, *HTN* hypertension, *DM* diabetes mellitus, *CHF* congestive heart failure, *CAD* coronary artery disease, *PVD* peripheral vascular disease, *CVA* cerebrovascular event, *AF* atrial fibrillation, *ESRD* end stage renal disease, *GN* glomerulonephritis

^a^Characteristics shown as *n* (%), except for age, which is shown as mean ± 1 standard deviation

^b^983 (8.9 %) missing values for One Stroke, and 93 (9.6 %) missing values for > 1 Stroke

### The multistate model: factors associated transitions to future ischemic stroke and to death

The results of the multistate model, which by design models both factors associated with subsequent strokes and survival following (separately) a first or subsequent stroke, are shown in Table 2; this model is adjusted for all factors listed in Table 1. Increasing age was associated with likelihood of transitioning from either a first (per-decade AHR 1.26, 95 % CIs 1.24 – 1.29, *P* < 0.0001) or second (1.40, 1.30 – 1.50, *P* < 0.0001) stroke to death, while female sex was associated with an increased risk of having a subsequent stroke (1.37, 1.20 – 1.56, *P* < 0.0001). African-Americans, as compared to Caucasians, had lower likelihood of dying after a first stroke (0.81, 0.77 – 0.85, *P* < 0.0001). A low BMI (<20 kg/m^2^) was associated with an increased likelihood of dying after a first stroke (1.19, 1.11 – 1.28, *P* < 0.0001), while higher BMI categories (25 – 29.99 kg/m^2^ and ≥ 30 kg/m^2^) were associated with a lower risk of death after either first (0.91, 0.86 – 0.97, *P* = 0.0012 and 0.72, 0.61 – 0.86, *P* = 0.0002, respectively) or second (0.92, 0.87 – 0.97, *P* = 0.0038 and 0.80, 0.67 – 0.96, *P* = 0.018, respectively) stroke. Diabetics were at increased risk of death after a first stroke (1.07, 1.02 – 1.12, *P* = 0.0070).

**Table 2:** Adjusted hazard ratios of covariates for the multistate model

	AHR	95 % CI’s	*P-*Value
Age (per decade) _stroke 1→ death_	1.26	1.24 – 1.29	<0.0001
Age (per decade) _stroke 2→ death_	1.40	1.30 – 1.50	<0.0001
Female sex _stroke 1→ stroke 2_	1.37	1.20 – 1.56	<0.0001
Race^a^ AA _stroke 1→ death_	0.81	0.77 – 0.85	<0.0001
Race^a^ Other _stroke 2→ death_	0.55	0.34 – 0.88	0.013
BMI^b^ <20 kg/m^2^_stroke 1→ death_	1.19	1.11 – 1.28	<0.0001
BMI^b^ 25–29.9 kg/m^2^_stroke 1→ death_	0.91	0.86 – 0.97	0.0012
BMI^b^ 25–29.9 kg/m^2^_stroke 2→ death_	0.72	0.61 – 0.86	0.0002
BMI^b^ ≥30 kg/m^2^_stroke 1→ death_	0.92	0.87 – 0.97	0.0038
BMI^b^ ≥30 kg/m^2^_stroke 2→ death_	0.80	0.67 – 0.96	0.018
Diabetes _stroke 1→ death_	1.07	1.02 – 1.12	0.0070
CHF _stroke 1→ death_	1.19	1.14 – 1.24	<0.0001
PVD _stroke 2→ death_	1.29	1.09 – 1.55	0.0044
Previous stroke^c^_stroke 1→ stroke 2_	1.13	1.00 – 1.29	0.052
Previous stroke^c^_stroke 1→ death_	0.54	0.52 – 0.57	<0.0001
Previous stroke^c^_stroke 2→ death_	0.62	0.54 – 0.72	<0.0001
Smoker _stroke 2→ death_	2.14	1.50 – 3.03	<0.0001
Unemployed _stroke 1→ death_	1.35	1.10 – 1.66	0.0040
Inability to amb _stroke 1→ death_	1.22	1.09 – 1.36	0.0004
Inability to trans _stroke 1→ death_	1.22	1.03 – 1.44	0.021
Subsequent stroke _stroke 1→ death_^d^	1.72	0.96 – 3.09	0.071

*Abbreviations*: *AA* African-American, *BMI* body mass index, *CHF* congestive heart failure, *PVD* peripheral vascular disease, *amb* ambulate, *trans* transfer

^a^Reference category for race is caucasians

^b^Reference category for BMI is 20–24.9 kg/m^2^

^c^Represent a stroke prior to cohort inclusion, specifically a stroke before day 90 of dialysis

^d^Represents the risk of progressing to death via a second stroke versus progressing to death after only having had one stroke after entering the cohort. Thus, represents the adjusted hazard ratio for death of the second stroke compared to the first

A stroke prior to cohort entry tended to be associated with increased likelihood of experiencing a subsequent stroke (1.13, 1.00 - 1.29, *P* = 0.052), although previous strokes prior to cohort entry (e.g., during the predialysis period) were associated with lower likelihood of transitioning to death after either first or second strokes (0.54, 0.52 - 0.57, *P* < 0.0001 and 0.62, 0.54 - 0.72, *P* < 0.0001, respectively). After adjustment for all factors listed in Table 2, a subsequent stroke trended towards having a higher likelihood of resulting in transitioning to death compared to a first new stroke (1.72, 0.96 - 3.09), but this was of only borderline statistical significance (*P* = 0.071). In a sensitivity analysis in which we eliminated all individuals who experienced a stroke in the first 90 days after dialysis initiation (that is, before they satisfied criteria to be in the analytic cohort), results trended in the same direction, with the likelihood of transitioning to death compared to a first new stroke being 1.58 (0.74 - 3.39, *P* = 0.24).

### Temporal changes in the probabilities of transitioning from first ischemic stroke to a subsequent ischemic stroke or death

**Fig. 3:**
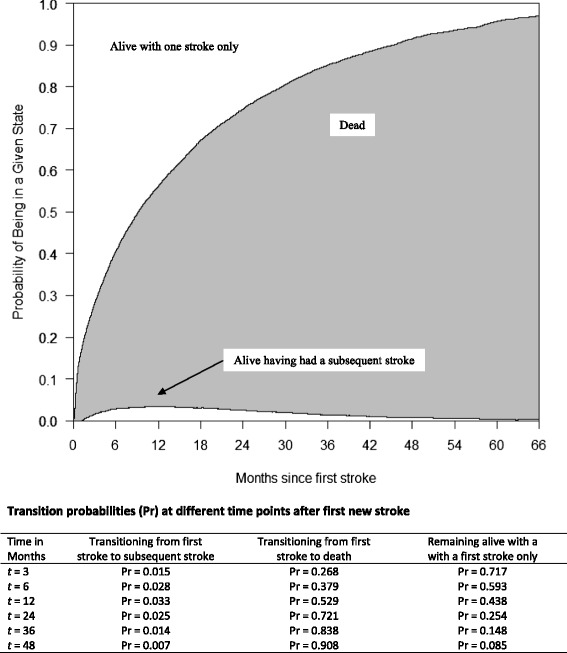
Transition probability graph for stroke

A “stacked transition probability graph” is shown in Fig. 3, which demonstrates the probability of transitioning between different states (e.g., from first stroke to subsequent stroke, from first stroke to death, and remaining alive after having had only 1 stroke); a table of transition probabilities between states, shown as time elapses after first new stroke, accompanies the figure. As can been seen, probability of transition to death increased as time elapses, as expected. However, the probability of transitioning to a subsequent stroke increased most sharply over the first 6 months, rose more slowly until reaching a peak at approximately 12 months, then declined steadily as time elapses.

### Timing of a subsequent ischemic stroke and resultant survival probabilities

**Fig. 4:**
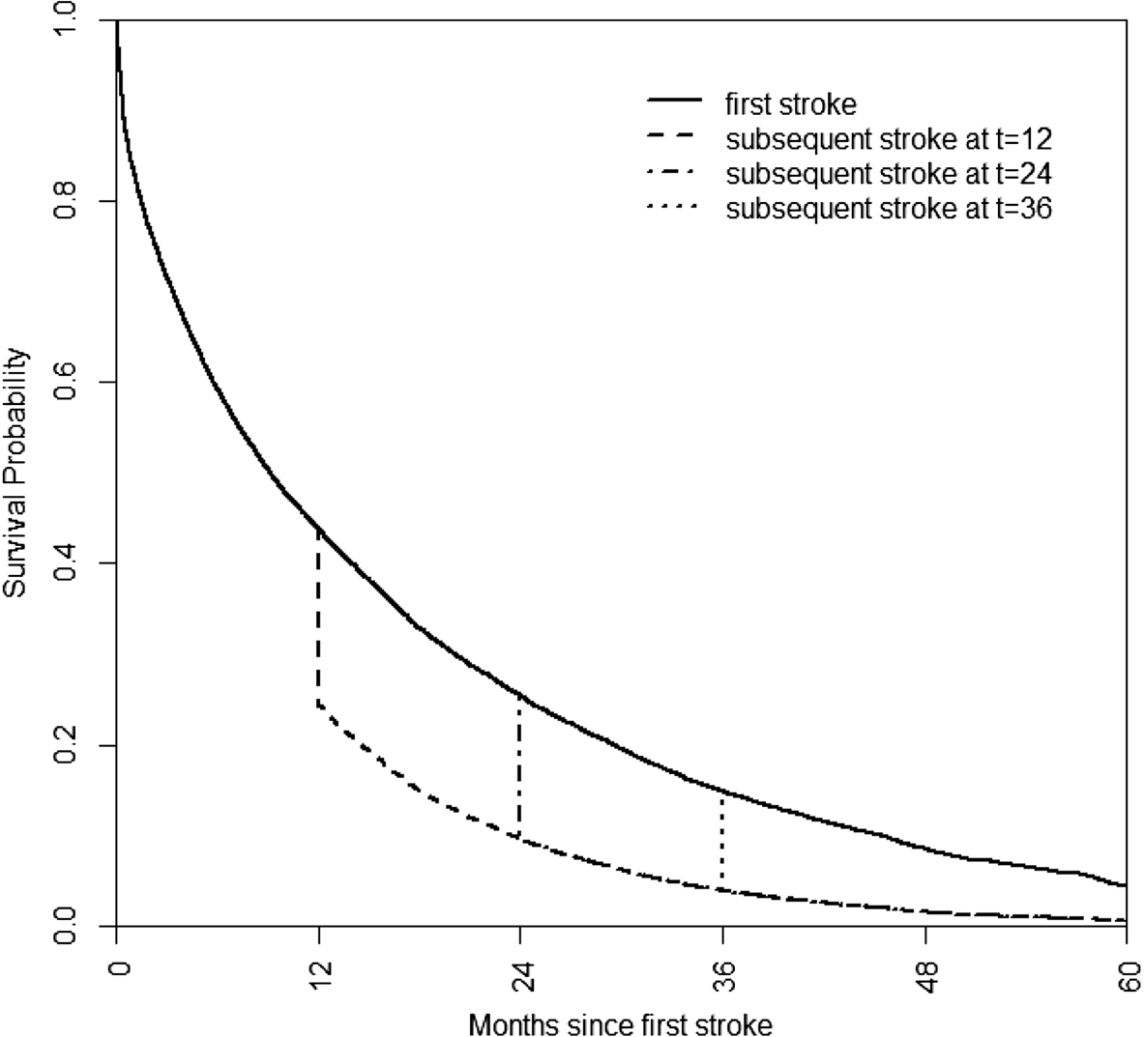
Survival plot after first and subsequent strokes

The effect of a subsequent stroke, generated for the sample-based average risk profile for the cohort, is shown in another way in Fig. 4, which demonstrates the impact on survival of a subsequent stroke. The initial survival curve (that representing survival after a first stroke) generates a trajectory, the change of which is then modelled and demonstrated by a subsequent stroke which occurs at a subsequent time; 12, 24, and 36 months have been selected for demonstration purposes. When a subsequent stroke occurred at 24 months, probability of survival decreased >15 %, in absolute terms, from 0.254 to 0.096. A substantial drop was seen at subsequent time points (e.g., 36 months), in each case leaving less than half of the remaining probability of survival.

## Discussion

In this study, we examined recurrent strokes in patients receiving chronic dialysis. In patients who had experienced a first stroke on dialysis, we sought to determine what factors were associated with subsequent strokes and to assess the relative hazard for death of a subsequent stroke compared to an initial one. Since patients with an initial stroke can remain alive without a subsequent stroke, experience a subsequent stroke, or die after either of a first or subsequent stroke, we reasoned that a multistate model was a suitable approach to investigate these questions. Our principal findings were that females had a substantially increased risk of subsequent stroke compared to males; that African-Americans had greater survival after a first new stroke than Caucasians; that the likelihood of experiencing a second stroke, compared to remaining alive after a first new stroke or dying, increases rapidly over the first 6 months but later diminishes; and that a subsequent stroke markedly decreases survival probability and tends to be associated with increased risk of mortality relative to a first new stroke.

The issue of subsequent or multiple stokes has not been well-addressed in the dialysis literature; indeed, data is somewhat sparse even in the nondialysis population [19]. While a substantial body of work has been compiled regarding stroke in dialysis patients [3–10, 20–23], the relative effects of subsequent strokes appears not to have been specifically studied. The overall number of subsequent strokes as relatively low, perhaps because of the high mortality associated with an index stroke [10]. However, with prevalent dialysis patients living substantially longer, on average, than was the case just a few years ago [2], this issue of recurrent stroke may take on increasing importance, especially since stroke is strongly related to increasing age [24].

That females on dialysis were substantially more likely to experience a subsequent ischemic stroke than males has not, to our knowledge, been specifically reported. However, insights can be garnered from other work in both the dialysis and non-dialysis patients populations. While some studies have suggested that there is no difference in HR for stroke between males and females [3, 6], we have previously found females to be at higher risk of ischemic stroke. Additionally, Power et al. reported the HR for stroke among females to be approximately 1.25 [8], while Seliger et al. found females to be more likely to experience a stroke in univariate (HR 1.33), but not multivariable analyses [5]. Recently, the important role that sex differences might play in stroke has received increased scrutiny. An analysis by Paulus et al. [25] suggested that sex differences appear to be important in risk of ischemic stroke and in post-stroke survival in the general population. While they found that females had a reduced risk of mortality after an ischemic stroke, we observed no such differences (although females had an increased risk of incurring a subsequent ischemic stroke). It may be that epidemiological findings in the general population, such as those relating to sex, may not be generalizeable to the HD population, as the latter have both quantitative and qualitative differences in stroke risk factors compared to the former.

Another major demographic finding concerned race. Compared to African-Americans, Caucasians were more likely to die following a first new ischemic stroke. The issue of survival on dialysis is a complex one, with recent work suggesting that, at least among older individuals (who also comprise those most likely to experience major cardiovascular events such as stroke), African-Americans had better survival than Caucasians [26]. The improved survival of racial minorities has also been demonstrated in other HD populations, such as that of the UK [27–29]. Racial differences in stroke incidence may also be related to previous disease burden, since Seliger et al. reported that differential stroke risk by race interacts with the presence of previous cardiovascular disease [5]. If true, this suggests that there are complexities in stroke epidemiology by race in dialysis patients. Our findings seem to indicate that African-Americans may survive strokes that Caucasians do not. Whether this might be due to biological differences, phenotypic differences in stroke by race, stroke treatment, or other factors is unknown. More broadly, this phenomenon may be operative for other cardiovascular events such as myocardial infarctions or complications of peripheral vascular disease; if so, the greater ability of black, as opposed to white, HD patients to weather catastrophic cardiovascular events may be a reason for greater longevity in black, as compared to white, HD patients.

Subsequent ischemic strokes demonstrated a noteworthy temporal pattern. Multistate models have the advantage of permitting risks to “compete” against each other by generating relative transition probabilities at varying times. This permits inferences to be made about important clinical questions, such as whether a subsequent stroke changes the risk of progression to death compared to having only one stroke. We found that the transition probability to a subsequent ischemic stroke increased over time (particularly during the first 6 months after an initial stroke), peaked at 12 months, and then declined. Within the framework of the multistate model, this later decline is likely the result of an increasing probability of transitioning to death. However, our findings suggest that, among those who do survive, risk of subsequent stroke may ultimately begin to decline as time from initial stroke increases.

We were initially uncertain as to the relative effect on mortality of a subsequent, as compared to a first new, ischemic stroke. While the survival plots demonstrated, as would be expected, that a subsequent stroke has a marked effect of decreasing survival (an effect which increased proportionately as time from the initial stroke elapsed), this does not directly address the issue of relative risk of mortality of first, as compared to subsequent, strokes. We did find a signal that a subsequent stroke may confer a greater risk of mortality compared to the first by ≈ 70 %. However, the odds ratio narrowly missed the traditional threshold of statistical significance, likely due to relatively modest number of subsequent strokes, thus definitive conclusions con not be drawn.

These findings must be appreciated within the context of an apparent paradox suggested by the HRs of < 1 for patients with a history of stroke (within, or even before, the first 90 days of dialysis initiation) associated with the transition of ischemic stroke to death. This unexpected finding may be the result of competition with death during the first critical 90 days after dialysis initiation: since the sickest patients are likely the ones who die soonest, only the healthiest or most resilient are likely to survive the first 90 days to become observable. Because death is, in effect, an outcome “competing” with a stroke, a stroke within the first 90 days of dialysis (or even before dialysis initiation) appears, upon superficial examination, to be “protective” for the transition from stroke to death.

## Conclusions

This may be the first report on the impact of recurrent strokes on survival in dialysis patients. However, our findings should be interpreted in the context of important limitations. First, our outcomes were based upon claims, so specific stroke characteristics such as the size or severity of the stroke are unknown. As a claims-based analysis, we could study only events which resulted in an encounter with the healthcare system; strokes that were clinically silent could not be examined. It is therefore likely that a great many strokes which did not result in overt symptoms are not accounted for in our analysis. Our claims-based approach also means that patient-level measures such as blood pressures or laboratory values were not available. Second, the number of subsequent strokes, relative to the number of first strokes, was modest. Thus, the effect of subsequent, as compared to a first new, stroke on mortality demonstrated wide confidence intervals which crossed unity at the lower bound. This limits the ability to draw “definitive” conclusions, and so our results should be considered hypothesis-generating. Third, we do not have detailed knowledge of changes in treatment which result after a stroke. It could be possible, for example, that females receive different types of post-stroke interventions than males, or that African-Americans are treated differently than Caucasians. Thus, our findings may be the result of differences in treatment in various clinical scenarios rather than any inherent differences by sex or race. Another limitation is the paradox that can result from an index event bias, in which risk factors which predispose for an initial event appear to be “protective” against recurrent events [30]. This phenomenon can occur when there is congruence between the risk factors for the index and recurrent events, and can result in an underestimation of a the effect of risk factor on subsequent events [31]. As such, our estimates of the effect of a previous stroke on death following a subsequent stroke may not be robust; caution should be used when making inferences about the importance of the point estimates. Nonetheless, confidence in our findings is bolstered by the fact that our modeling approach resulted in several expected associations, increasing face validity. For example, increasing age (per decade) was associated with death after a first or subsequent stroke, high BMI appeared to be “protective” in nature, and a diagnosis of diabetes was associated with death following a first new ischemic stroke. Generalizability may be limited to US dialysis patients, particularly those who use Medicare as their primary payer. Finally, we did not censor patients if they transitioned to peritoneal dialysis, but change in modality is likely to occur only in a very modest number of US hemodialysis patients, since when used, peritoneal dialysis is typically employed as an initial, or “index”, therapy, in the US.

In summary, females are more likely to experience subsequent strokes than males, and Caucasians are more likely to die after a first new ischemic stroke than African-Americans. If the latter finding were to be replicated in other cardiovascular disorders, it might partially explain the phenomenon of older African-Americans have better survival on dialysis than Caucasians. The risk of a transitioning to a subsequent stroke, after having had a first, demonstrates a pattern of increase until about 1 year, followed by decrease which is likely the result of “competing” with the outcome of death. Subsequent strokes may be associated with a substantial decrease in survival probability, a relative effect which increases as time since first stroke elapses. This information may be of assistance to clinicians when inform hemodialysis patients about the implications of recurrent stroke, but more work is needed.

## References

[1] Berry JD, Dyer A, Cai X, Garside DB, Ning H, Thomas A, Greenland P, Van Horn L, Tracy RP, Lloyd-Jones DM (2012). Lifetime risks of cardiovascular disease. N Engl J Med.

[2] United States Renal Data System (2012). USRDS 2012 Annual Data Report: Atlas of End-Stage Renal Disease in the United States. In: National Institutes of Health, National Institute of Diabetes and Digestive and Kidney Diseases.

[3] Wiesholzer M, Harm F, Tomasec G, Barbieri G, Putz D, Balcke P (2001). Incidence of stroke among chronic hemodialysis patients with nonrheumatic atrial fibrillation. Am J Nephrol.

[4] Seliger SL, Gillen DL, Longstreth WT, Kestenbaum B, Stehman-Breen CO (2003). Elevated risk of stroke among patients with end-stage renal disease. Kidney Int.

[5] Seliger SL, Gillen DL, Tirschwell D, Wasse H, Kestenbaum BR, Stehman-Breen CO (2003). Risk factors for incident stroke among patients with end-stage renal disease. J Am Soc Nephrol.

[6] Sozio SM, Armstrong PA, Coresh J, Jaar BG, Fink NE, Plantinga LC, Powe NR, Parekh RS (2009). Cerebrovascular disease incidence, characteristics, and outcomes in patients initiating dialysis: the choices for healthy outcomes in caring for ESRD (CHOICE) study. Am J Kidney Dis.

[7] Sanchez-Perales C, Vazquez E, Garcia-Cortes MJ, Borrego J, Polaina M, Gutierrez CP, Lozano C, Liebana A (2010). Ischaemic stroke in incident dialysis patients. Nephrol Dial Transplant.

[8] Power A, Chan K, Singh SK, Taube D, Duncan N (2012). Appraising stroke risk in maintenance hemodialysis patients: a large single-center cohort study. Am J Kidney Dis.

[9] Wetmore JB, Ellerbeck EF, Mahnken JD, Phadnis MA, Rigler SK, Spertus JA, Zhou X, Mukhopadhyay P, Shireman TI (2013). Stroke and the “Stroke Belt” in Dialysis: Contribution of Patient Characteristics to Ischemic Stroke Rate and Its Geographic Variation. J Am Soc Nephrol.

[10] Wetmore JB, Phadnis MA, Ellerbeck EF, Shireman TI, Rigler SK, Mahnken JD (2015). Relationship between stroke and mortality in dialysis patients. Clin J Am Soc Nephrol.

[11] Wetmore JB, Rigler SK, Mahnken JD, Mukhopadhyay P, Shireman TI (2010). Considering health insurance: how do dialysis initiates with Medicaid coverage differ from persons without Medicaid coverage?. Nephrol Dial Transplant.

[12] Volkova N, McClellan W, Soucie JM, Schoolwerth A (2006). Racial disparities in the prevalence of cardiovascular disease among incident end-stage renal disease patients. Nephrol Dial Transplant.

[13] Andrade SE, Harrold LR, Tjia J, Cutrona SL, Saczynski JS, Dodd KS, Goldberg RJ, Gurwitz JH (2012). A systematic review of validated methods for identifying cerebrovascular accident or transient ischemic attack using administrative data. Pharmacoepidemiol Drug Saf.

[14] Go AS, Hylek EM, Chang Y, Phillips KA, Henault LE, Capra AM, Jensvold NG, Selby JV, Singer DE (2003). Anticoagulation therapy for stroke prevention in atrial fibrillation: how well do randomized trials translate into clinical practice?. Jama.

[15] Putter H, Fiocco M, Geskus RB (2007). Tutorial in biostatistics: competing risks and multi-state models. Stat Med.

[16] Klein JP, Shu Y (2002). Multi-state models for bone marrow transplantation studies. Stat Methods Med Res.

[17] de Wreede LC, Fiocco M, Putter H (2010). The mstate package for estimation and prediction in non- and semi-parametric multi-state and competing risks models. Comput Methods Programs Biomed.

[18] Klein JP, Keiding N, Copelan EA (1993). Plotting summary predictions in multistate survival models: probabilities of relapse and death in remission for bone marrow transplantation patients. Stat Med.

[19] Kamel H, Johnson DR, Hegde M, Go AS, Sidney S, Sorel M, Hills NK, Johnston SC (2012). Detection of atrial fibrillation after stroke and the risk of recurrent stroke. J Stroke Cerebrovasc Dis.

[20] Wetmore JB, Phadnis MA, Mahnken JD, Ellerbeck EF, Rigler SK, Zhou X, Shireman TI (2014). Race, Ethnicity, and State-by-State Geographic Variation in Hemorrhagic Stroke in Dialysis Patients. Clin J Am Soc Nephrol.

[21] Toyoda K, Fujii K, Fujimi S, Kumai Y, Tsuchimochi H, Ibayashi S, Iida M (2005). Stroke in patients on maintenance hemodialysis: a 22-year single-center study. Am J Kidney Dis.

[22] Iseki K, Fukiyama K (1996). Predictors of stroke in patients receiving chronic hemodialysis. Kidney Int.

[23] Iseki K, Fukiyama K (2000). Clinical demographics and long-term prognosis after stroke in patients on chronic haemodialysis. The Okinawa Dialysis Study (OKIDS) Group. Nephrol Dial Transplant.

[24] Mozaffarian D, Benjamin EJ, Go AS, Arnett DK, Blaha MJ, Cushman M, de Ferranti S, Despres JP, Fullerton HJ, Howard VJ, Huffman MD, Judd SE, Kissela BM, Lackland DT, Lichtman JH, Lisabeth LD, Liu S, Mackey RH, Matchar DB, McGuire DK, Mohler ER, Moy CS, Muntner P, Mussolino ME, Nasir K, Neumar RW, Nichol G, Palaniappan L, Pandey DK, Reeves MJ, Rodriguez CJ, Sorlie PD, Stein J, Towfighi A, Turan TN, Virani SS, Willey JZ, Woo D, Yeh RW, Turner MB (2015). American Heart Association Statistics C, Stroke Statistics S: Heart disease and stroke statistics--2015 update: a report from the American Heart Association. Circulation.

[25] Paulus JK, Lai LY, Lundquist C, Daneshmand A, Buettner H, Lutz JS, Raman G, Wessler BS, Kent DM. Field Synopsis of the Role of Sex in Stroke Prediction Models. J Am Heart Assoc. 2016;5(5). [epub ahead of print]. 10.1161/JAHA.115.002809 PMC4889171 27151514

[26] Kucirka LM, Grams ME, Lessler J, Hall EC, James N, Massie AB, Montgomery RA, Segev DL (2011). Association of race and age with survival among patients undergoing dialysis. JAMA.

[27] Roderick P, Byrne C, Casula A, Steenkamp R, Ansell D, Burden R, Nitsch D, Feest T (2009). Survival of patients from South Asian and Black populations starting renal replacement therapy in England and Wales. Nephrol Dial Transplant.

[28] Wagner M, Ansell D, Kent DM, Griffith JL, Naimark D, Wanner C, Tangri N (2011). Predicting mortality in incident dialysis patients: an analysis of the United Kingdom Renal Registry. Am J Kidney Dis.

[29] Udayaraj U, Pruthi R, Casula A, Roderick P (2013). UK Renal Registry 16th annual report: chapter 6 demographics and outcomes of patients from different ethnic groups on renal replacement therapy in the UK. Nephron Clin Pract.

[30] Kent DM, Thaler DE (2010). Is patent foramen ovale a modifiable risk factor for stroke recurrence?. Stroke.

[31] Dahabreh IJ, Kent DM (2011). Index event bias as an explanation for the paradoxes of recurrence risk research. JAMA.

